# Digital integrated dramatherapy: A feasibility study in women undergoing assisted reproductive technology

**DOI:** 10.3389/fpsyg.2022.1045090

**Published:** 2022-12-16

**Authors:** Maria Cecilia Cercato, Sandra Pierpaoli, Rosa Maria Pazienza, Irene Terrenato, Cinzia Guadagnuolo, Cristina Cenci, Rossella E. Nappi

**Affiliations:** ^1^Epidemiology and Cancer Registry Unit, IRCCS–Regina Elena National Cancer Institute, Rome, Italy; ^2^CDI narrAZIONI, Rome, Italy; ^3^Clinical Trial Center, Biostatistics and Bioinformatics, IRCCS – Regina Elena National Cancer Institute, Rome, Italy; ^4^Digital Narrative Medicine (DNM), Rome, Italy; ^5^Department of Clinical, Surgical, Diagnostic and Pediatric Sciences, University of Pavia, Pavia, Italy; ^6^Unit of Reproductive Medicine, Gynecological Endocrinology and Menopause, IRCCS–S. Matteo Foundation, Pavia, Italy

**Keywords:** arts therapies, creativity, assisted reproductive technology, narration, corporeal expression

## Abstract

**Background:**

Dramatherapy is a practice of working and playing that uses action methods to facilitate creativity, imagination, learning, insight and growth.

**Methods:**

A pilot study of Digital Integrated Dramatherapy, recruiting women from the digital community “Parole Fertili,” undergoing assisted reproductive technology. On the basis of a previous blended experience, a program based on remote sessions was conducted on a dedicated platform.

**Results:**

A total of 22 women participated in the same intervention in three groups. Participants assessed the feasibility and utility of the method, both in the synchronous and asynchronous phases. The group had a fundamental role: the participants were supportive, and therapeutic benefits were due to strengthening and resilience obtained through a dialogue with other women. Using metaphors, the participants could move from the narration of the Assisted Reproductive Technology pathway to creative and corporeal expression.

**Conclusion:**

The study showed that a group based on Digital Integrated Dramatherapy might help women face very difficult emotions by promoting creativity and internal resources.

## Introduction

The key messages of a recent WHO report were that integrating play with other arts provides health benefits through several components, including aesthetic commitment, imagination involvement, sensory activation, induction of emotion, and cognitive stimulation, and that validated models for the implementation in clinical, academic and education settings are necessary (Fancourt and Finn, 2019).

Creativity may be described as the ability to produce answers to the world by changing it through one’s solutions. It is not restricted to a limited group of individuals, but it is present in everyone independently of age, mental and physical condition. It is expressed by children in play and by adults in arts (Winnicott, 1993). In addition, according to the Infant Research hypotheses, a drama ability is inborn and takes part in the mother/newborn dialogue through body expressivity. These early conversation exchanges build self, body experience, and relationships with the world (Stern, 1998). Creativity is an essential resource for individual and collective well-being and may contribute to health when developed in a curative setting.

According to the British Association of Dramatherapists, Dramatherapy is a practice of working and playing that uses action methods to facilitate creativity, imagination, learning, insight and growth (British Association of Dramatherapists, 2022). In particular, Integrated Dramatherapy (ID) is a specific methodology developed and practiced at the narrAZIONI Integrated Dramatherapy Center (CDInarrAZIONI, 2022). It uses all languages expression, both verbal and not verbal, cooperating with the structure of drama representation. This activity may induce creative processes during which the subject can perceive, represent and recount themselves or parts of themselves, promoting an easier circulation of all the person’s aspects.

Promoting creativity may activate sensory channels that reconnect the person with their internal potential; it may strengthen the confidence in completing a constructive path, starting from imagination and self-perception and up to realization in expression and representation. Thanks to these characteristics, creativity may be an important resource for women undergoing assisted reproduction technology (ART). The journey through ART is considered a special condition, including a life crisis and possibly remodeling psychosocial and relationship variables (Nappi et al., 2004). These variables could contribute to medical outcomes and physical as well as emotional health after the medical procedure (Rooney and Domar, 2016). Infertility is a common condition; 20% of Italian couples of childbearing age have reproductive issues. The Italian Registry of Italian Health Institute reported that around 80,000 ART cycles were performed in 2020. Only 20% of them had a positive outcome. These data show that the problem is common and suggest that the care pathway is complex and uncertain (Scaravelli et al., 2021).

This study is part of a wider digital narrative project called Parole Fertili (Fertile Words). This project started in 2016 as a digital community centered on story sharing. It was created by a multidisciplinary group of anthropologists, psychologists, writers, and experts in digital media, with the unconditioned support of the IBSA Foundation for scientific research. Hundreds of stories about the quest for a child were shared on the website (Parole Fertili web, 2021), and the web community gathered more than 20,000 followers on Facebook (Parole Fertili Facebook, 2021). The website used different language and activities, including video storytelling and theatre. A total of 50 of these stories were published in a book that describes ambivalence, needs, desires, and fears associated with fertility issues and serves as a supportive tool in ART centers, facilitating understanding and sharing of experiences (Cenci and Dimitri, 2018).

The analysis of stories from the community suggested that narrative and shared creativity might be relevant in psychological support. The results provided the basis for evaluating the Digital Integrated Dramatherapy (DID) path as an original methodology in this population. A digital narrative methodology was previously demonstrated to be adequate in different clinical conditions (Volpe and Testa, 2019; Cenci and Mecarelli, 2020; Cercato et al., 2022a,b). The digital platform used in this project was specifically designed for narrative medicine and psychological support. It was evaluated as a digital narrative diary (DNMLab) tool in oncology, cardiology, and neurology (Volpe and Testa, 2019; Cenci and Mecarelli, 2020; Cercato et al., 2022a,b). In addition, the digital platform [PSYDIT (Psychology Dital Tools), n.d.] has been used for psychological support in several scenarios, such as during an emergency due to the COVID-19 pandemic (Giusti et al., 2020).

The usability of ID for psychological support was first tested in 2019 in the pilot study “Creativity as a resource for the journey in tumor” carried out in the IRCCS – Regina Elena National Cancer Institute targeting women affected by breast cancer and followed by the Psychology Unit of the Regina Elena Institute. It consisted of a series of experiences with laboratories for body expression based on the use of artistic languages. It was prematurely closed after the first laboratory due to the restrictions of the COVID-19 pandemic. Nevertheless, this experience allowed the validation of tools for the outcomes assessments (the clinical records and the self-assessment questionnaires) and the introduction of a digitally based pathway with multimedia prompts to use creativity for exploration, self-awareness, and interaction.

Several studies highlighted the effectiveness of Dramatherapy in different areas of health, such as schizophrenia, eating disorders, and oncological diseases, involving different target populations, such as children, adolescents, the elderly, and couples; however, they mainly refer to the use of the theatrical medium as a valid tool for therapeutic intervention (Chasin et al., 1989; Bielańska et al., 1991; Ruddy and Dent-Brown, 2007; Knox and Svendsen, 2015; Keisari and Palgi, 2017; Purrezaian et al., 2020). Instead, the present study focuses on an innovative methodology, the Integrated Dramatherapy applied to digital, in which the use of artistic languages is combined with psycho-corporeal techniques, with the aim to bring participants to the construction of a metaphorical path by integrating bodily movements, pictures research and production, figurative and plastic creation, reflective and creative writing, dramatization and verbalization (CDInarrAZIONI, 2022).

No published studies were found from a systematic review of literature aiming to explore the role of Arts Therapies and Dramatherapy in women involved in ART conducted by the Authors (Supplemental material). Therefore, this innovative feasibility study was designed to serve as the basis for validating an intervention methodology in the ART setting.

The study’s first aim was to assess the feasibility of a digital multimedia Dramatherapy pathway for psychological support to women experiencing ART and attending the Parole Fertili community.

A secondary aim was to evaluate the usefulness of DID for awareness of one’s experiences, self-representation and the self-narrative through arts languages, and analysis of experience with ART.

## Research approach and methodology

### Study design

This was a preliminary, open, uncontrolled study in a real-life setting, designed to explore the role of an innovative clinical methodology for Digital Integrated Dramatherapy, DID, as a supportive care tool for women involved in ART and part of the community Parole Fertili. A qualitative descriptive methodology was used to described the reactions and the opinion of the participants during the intervention through: (a) direct observation (the observer); (b) participatory observation (the researcher, defined as “conductor,” is part of the situation being studied); (c) analysis of documents (texts, pictures, multimedia contents) (Luciani et al., 2019). A quantitative methodology was also used (self-evaluation questionnaires, clinical sheets) in order to assess outcome parameters (applicability) expressed by standard score (see Outcome Assessment section).

This was an 8-month study, including the following phases: enrollment, organization of participants groups and registration on PSYDIT (over 2 months); DID pathway and data recording (over 3 months); data analysis (over 3 months).

### Participant population and research context

Participating in creating health content through blogging and social networking plays a consistent role in patients’ health experiences (Ziebland and Wyke, 2012). Blogging practices of women undergoing IVF is an emerging communication channel that can offer a more complete picture of the IVF experience in a real-life setting (Orr et al., 2017). According to these criteria in the present study, the chosen site was virtual, and the sampling was online. Advantages of internet recruitment include geographic diversity and greater access to marginalized groups (Hamilton and Bowers, 2006).

The project was launched through the digital community Parole Fertili and enrolled Italian-speaking women aged ≥18 years who had experienced or were undergoing ART with any outcome. Women were recruited through a call to action on the community’s social media (Facebook, Twitter, Instagram). Information on the pathway was available on a landing page. Women were included in the project after an individual phone interview and could register on PSYDIT upon invitation by a psychologist. Registration was free. Written informed consent to participate and confidential data handling procedures for research and assistance according to the Italian Law 193/2006 on Privacy and Safeguarding of Sensitive Data and the General Data Protection Regulation of the European Union, 2016/279 [Personal data code protection, Regulation (EU) 2016/679] was required [Personal data code protection, 2003, Regulation (EU) 2016/679 (Regulation (EU), 2016a,b)]. In particular, participants provided their consent to publish materials produced in the project.

The pilot study “Creativity as a resource for the journey in tumor” carried out in the IRCCS – Regina Elena National Cancer Institute was approved by the local Ethical Committee [DIPSO study: Prot. N. Registrazioni Sperimentali 1255/19(2265) 09.26.2019].

### Researcher team

The project team included health professionals trained in the application of Arts Therapies: a clinical psychologist and theater therapist (referred to as an observer) and psychologist, psychotherapist, and dramatherapist (referred to as a conductor), who is also the founder of the ID and DID method and of the Center for Integrated Dramatherapy (CDI) narrAZIONI (CDInarrAZIONI, 2022). Moreover, the team included an oncologist-epidemiologist, with expertise in methodology for a humanistic approach to medicine, an anthropologist, responsible for the study design and analysis who designed the PSYDIT platform and planned the Parole Fertili digital story-sharing community, a digital community manager, and a gynecologist with expertise in reproductive endocrinology and infertility.

### PSYDIT

The pathway of DID was carried out using the PSYDIT platform, which was developed by the startup DNM (DNM, 2022).

PSYDIT is a protected digital environment and includes tools for individual or group psychological support: expressive writing, narrative prompts, multimedia, evaluation questionnaires, and an area for the care team comments. Patient access to PSYDIT was granted only upon invitation by the psychologists. Each participant received the credentials to activate their account to enter their group area on the platform.

Both participants and researchers signed their consent to the use of PSYDIT and the confidential data handling procedures for research and assistance according to the current legislation. Participants scheduled activities, such as group meetings and personal tasks, were recorded in the platform calendar. Virtual interactions between participants and the healthcare team were made possible. All files used in the pathway were uploaded to the platform, and data were anonymized in CSV format for analysis.

### Digital integrated dramatherapy: The pathway for ART

DID is a new method conceived and experienced by the Center for Integrated Dramatherapy narrAZIONI, a clinical education and research center (CDInarrAZIONI, 2022). The method, which integrates arts and expressive therapies, is used by a group of specialized professionals: psychologists, psychotherapists, arts therapists, and physicians. DID is based on Bioenergetic Analysis, Arts Therapies, and the Theory of Dialogic Self. The Bioenergetic Analysis is a psychotherapeutic approach that views the psyche and soma as a functional unit so that events occurring in one reflect the other (Lowen, 2013a,b). Art Therapies include music, visual arts, dance, and theater, which are analogic languages deeply involved with our sensory channels (Kramer, 1977; Laban, 2003; Benenzon et al., 2005, 2006; Montanarella, 2006; Pitruzzella, 2014). Hermans and Dimaggio proposed the Theory of Dialogic Self in 2007 (Hermans and Dimaggio, 2007). According to this theory, the self includes several parts (voices, subjects, positions) with the possibility of dialogic relationships among each other (Hermans and Dimaggio, 2007).

The methodconsists of the conception and targeted construction of creative metaphoric pathways. Pathways may be carried out in person (ID), online (DID), or with blended (BID) presence/remote modality, depending on the type and needs of participants. Pathways are expressly prepared for each intervention and may support any phase of distress or disease.

Based on the narratives shared in the Parole Fertili community, the researchers prepared a metaphoric plot for the narrative phase of the project. The digital pathway was conceived and conducted by a conductor. It lasted 3 months and consisted of a synchronous phase of five biweekly video chat meetings, each lasting 2 h, and an asynchronous phase of the multimedia narrative. The pathway phases were (1) First synchronous video chat meeting, aimed at mutual knowledge and building of the group in the digital setting, where each participant introduced herself with a self-portrait. (2) Synchronous video chat: the psychotherapist held three meetings, each one dedicated to the conception, creation, and dramatization of a personalized mask (as detailed below). (3) Asynchronous phase: 13–15 multimedia narrative prompts on the chosen theme were administered to the participants every 3 days between chat meetings and at the end of the synchronous phase; the goal was for each participant to produce the narrative of their overall experience, which told the story of the DID journey. (4) A conclusive focus group video chat aimed at elaborating the individual path in the context and with the interaction of the group.

According to the methodology of descriptive qualitative studies, the video chat meetings were recorded, and an observer was in the group. Videorecording collection methods represent “*What Oka and Shaw (2000) refer to as “trustworthy” having “credibility, transferability, dependability, and confirmability,” which they say is “analogous to ‘internal validity’, ‘external validity’, ‘reliability’, and ‘objectivity’ in conventional criteria*” (Penn-Edwards, 2004). In this study, where a researcher followed subjects engaged in an activity, an observational recording method was used for evaluation, coding and interpretation of data. Recording during the video chat, moreover, prevented the bias of perceived intrusive use of equipment and personnel (Penn-Edwards, 2004).

The metaphoric plot was related to the different and ambivalent experiences of ART. Participants were taken to explore and share their own experience with ART through a tale entitled “Journey to the Land of Storytelling Masks,” based on metaphoric subjects along the pathway. Leverages were corporality, imagination, and creativity. Three masks were created and used to make parts of oneself dialogue together: The Talent (symbol of personal resources), The Restless Being (symbol of painful and ambivalent experiences), and The Queen (symbol of empowerment). The multimedia prompts of the asynchronous phase helped the participants develop the characteristics of the subjects and the experiences they represented. The final story was elaborated using images, photos, music, and descriptions, and the story was gradually developed.

**Figure 1:**
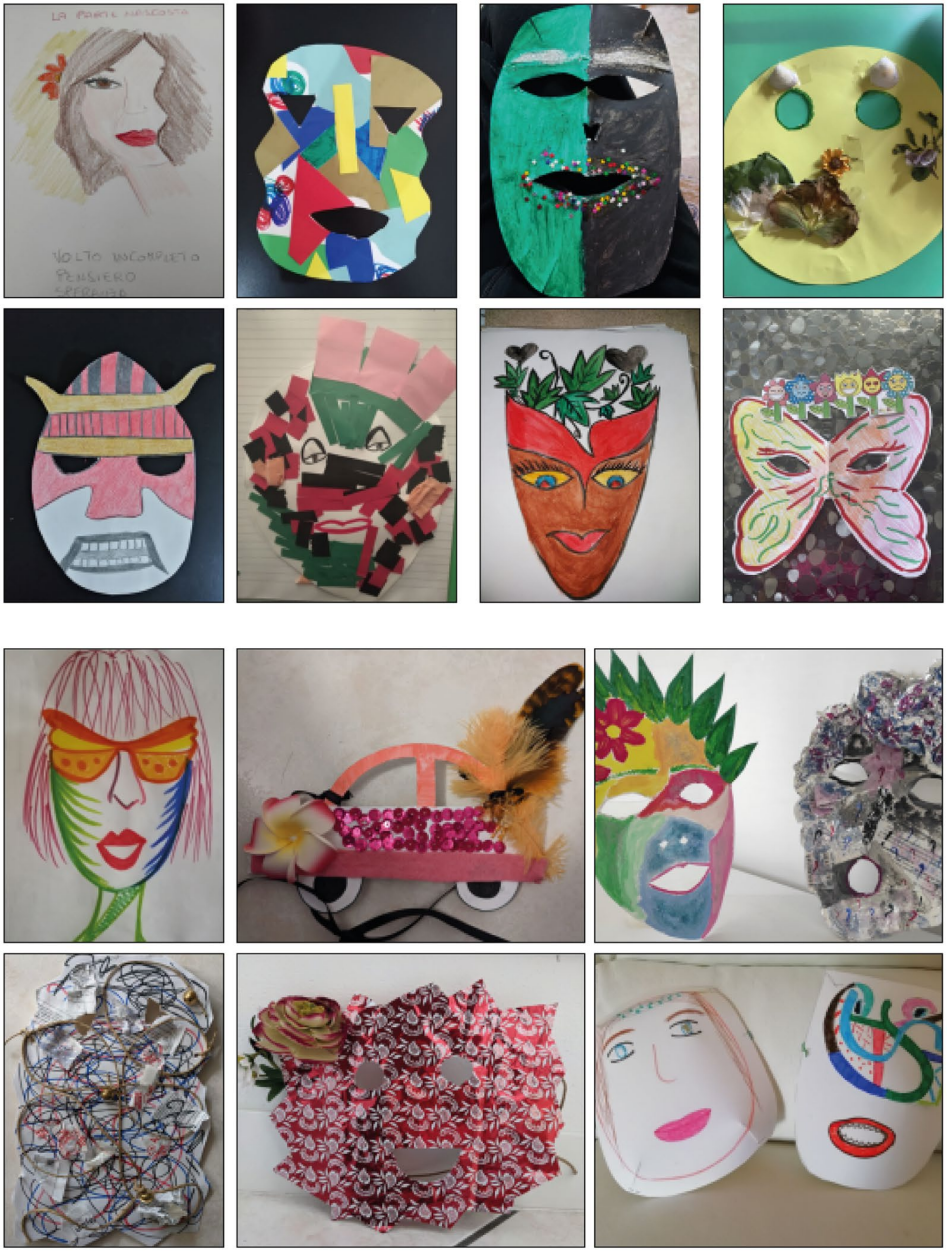
Artistic production of the synchronous phase.

Using metaphors with elements and subjects such as landscape, magic, fairy, chaos, and harmony favored the expression of experience with ART. Some actions were proposed during synchronous and asynchronous phases: corporeal and perceptive actions using short videos available on the platform and to be used as wanted; guided fantasies during the video-chat meetings and narrative prompts in the asynchronous phase; artistic actions such as drawing, mask production, creative movement, creative writing, and dramatization, during video-chat meetings (Figure 1); receptive and expressive actions using the search, the choice and the production of images and sounds in the asynchronous phase. A focus group was held in the last video chat allowing the participants to process the whole pathway, using key prompts suggested by the conductor, to develop aspects of the performances and get information on the experience (Migliorini and Rania, 2001).

The researcher team produced a final video for each group using PSYDIT to value the group’s work (Video a, 2021; Video b, 2021; Video c, 2021).

### Outcome assessment

The researchers chose feasibility and utility as markers to assess intervention outcomes. Parameters were individually evaluated by each participant and jointly by the conductor and the observer.

Data were recorded with (1) Self-evaluation questionnaires for expectations, with open and closed-ended questions, administered at the first access to the platform (Table 1). (2) Collection sheet of data concerning the dynamics observed in each group, elaborated by the psychologists based on clinical parameters, and filled in by the conductor with the help of the observer at all phases of the pathway: at the end of each meeting, during the asynchronous phase, and at the end of the focus group (Table 2). (3) Self-evaluation of participation, with open and closed-ended questions, administered to participants at the end of the pathway (Table 3). (4) Final board on the method applicability, filled in by psychologists for each group, at the end of the program, based on items presented in Table 4. Only those meetings with at least 35% of participants were considered. The group behavior was evaluated by observing participants and their interactions. Thematic areas of evaluation were behavior, participation, and productivity for the single participant; interest, climate, cohesion, alliance, interdependence, defense, and therapeutic benefit for groups (Table 2). Several parameters describe each thematic area. The presence and level of intensity of parameters were expressed by a score ranging from 1 (lowest) to 5 (highest). Each area was evaluated based on the mean of single scores. Comments were recorded.

**Table 1:** Answers to the initial questionnaire on participants’ expectations from the pathway (*n* = 18: multiple answers are allowed).

Question	Answer	*n* (%)
Previous experience with arts therapies	Yes	1 (5.55)
Aims of participation	Opportunity for sharing a common experience with women	6 (33.33)
	Support for expression and management of emotions related to ART	6 (33.33)
	Psychological support	5 (27.77)
Expected outcomes	Opportunity for sharing a common experience with women	8 (44.44)
	Increased sense of peace	5 (27.77)
	Increased awareness of oneself and emotions	4 (22.22)
	Opportunity for a dialogue with other women	3 (16.66)

**Table 2:** Health care professional’s assessment: results by group [score 1-5; area score (in bold) is the mean of parameter values].

**Indicators**		**Group A** *	**Group B** *	**Group C** *
**Areas**	**Parameters**	**Mean values (max 5)**	**Mean values (max 5)**	**Mean values (max 5)**
**Climate^**		**4.50**	**4.80**	**4.20**
	*Involvement*	4.50	5.00	3.80
	*Harmony*	4.50	4.60	4.60
**Cohesion^**		**4.50**	**3.87**	**3.87**
	*Membership*	3.50	3.60	3.60
	*Trust*	5.00	4.00	4.00
	*Safety*	5.00	4.00	4.00
**Alliance^**		**3.90**	**3.68**	**3.64**
	*Identification*	2.00	2.40	3.20
	*Empathy*	5.00	4.20	4.00
	*Tolerance*	4.00	3.80	4.00
	*Cooperation*	3.50	4.00	3.20
	*Solidarity*	5.00	4.00	3.80
**Interdependence^**		**4.00**	**4.08**	**3.57**
	*Realism*	5.00	2.80	3.20
	*Autonomy*	5.00	5.00	3.20
	*Acceptance*	3.50	3.80	3.60
	*Mirroring*	4.00	5.00	4.20
	*Resonance*	5.00	4.60	4.20
	*Individuation*	1.50	3.60	3.00
**Defense** ^**§**^		**1.19**	**1.20**	**1.15**
	*Criticism*	1.00	1.40	1.00
	*Role confusion*	1.00	1.00	1.00
	*Conflict displacement*	1.00	1.00	1.00
	*Attack and leak*	1.00	1.00	1.00
	*Scapegoat*	1.00	1.00	1.00
	*Attribution and projection*	1.00	1.00	1.00
	*Silences*	1.50	1.80	2.20
	*Escape to the outside*	2.00	1.40	1.00
**Therapeutic benefits^**		**2.68**	**3.65**	**3.30**
	*Self-disclosure*	4.00	3.60	3.40
	*Self_understanding*	2.50	3.40	3.00
	*Dynamicity*	2.50	3.60	3.00
	*Interpersonal learning*	2.50	3.60	3.00
	*Experimental*	2.00	3.00	3.00
	*Expression*	2.50	3.80	3.40
	*Introspection*	3.00	4.00	3.80
	*Symbolism*	2.50	4.20	3.80

* Group A met only two-times and it was not possible to evaluate the Focus Group; Group B and Group C met four-times and it was possible to evaluate the Focus Group;

§ Lower values stand for positive meaning.

^Higher values stand for positive meaning.

**Table 3:** Participants’ post-intervention self-assessment: feasibility and utility items (score 1–5).

Feasibility Items
The intervention length is adequate for aims
The digital tool is adequate for interactions with other participants and the conductor
The digital tool is adequate for introspection and imagination
The digital tool is adequate for self-expression and communication
The digital tool is adequate for experiencing an artistic language
Ease of use of the platform (synchronous phase)
Ease of use of the platform (asynchronous phase)
Ease of use of the platform (final production)
**Utility items**
Overall satisfaction with the whole pathway
Fulfillment of expectations
Adequacy to the phase of ART
Benefits from the interactions with the group

**Table 4:** Psychologists’ post-intervention assessment: applicability items (score 1–5).

**Items for the adequacy of the digital tool in the synchronous phase**
Duration of meetings in video chat
Consistency of women’s participation in the group
Quality of relationships with the group and the conductor
Usability of corporeal experiences
Usability of the artistic experimentation
Ease of use of the platform
Ease of management of the group
**Items for the adequacy of the digital tool in the asynchronous phase**
Number of multimedia digital prompts
Frequency of multimedia digital prompts
Response to multimedia digital prompts
Consistency of women’s participation in the group
Quality of relationships with the participants
Ease of use of the platform
Ease of management of the group
**Items of the overall pathway**
Adequacy of the intervention to aims

Feasibility parameters were evaluated by participants (Table 3) and psychologists (Table 4) with reference to the usability of the platform, the adequacy of the intervention duration and the digital tool for the DID pathway.

Researchers assessed utility based on markers of behavior, self-perception and awareness, relationship with space and time, relationship with others and the group, participation in the pathway and productivity, sensoriality features of the artistic production, abilities demonstrated during the drama explorations, response to multimedia narrative prompts, features of the individual final product, quality of interaction, and adequacy of the digital group.

Participant-based markers of utility (satisfaction, compliance to expectations according to the pathway phase, perceived benefit) are reported in Table 3.

Evaluating the applicability of DID on both feasibility and utility allowed the researchers to optimize the method and change the pathway when necessary.

### Statistical analysis

A database was recorded. Data were analyzed with a mixed quantitative/qualitative method. Numeric variables were quantitatively evaluated, while texts, open answers, and comments were analyzed qualitatively. Percentage, frequency distribution, mean, median, and range were calculated for quantitative analysis. Texts and multimedia content were used in this article to interpret quantitative data.

Due to the pilot nature of the study, an expected sample size of 28/30 patients was taken into account. Since this is a feasibility study, a conventional sample size estimate was not performed, mainly for two reasons: first, as reported by Luciani et al. (2019), “in qualitative research, sample size calculation is not used, and there are no strict rules to determine when to stop data collection”; second, based on the nature of the pilot study, we did not make any inference, so our study cannot be considered underpowered: we limited our quantitative analysis by reporting only descriptive statistics, as we mentioned in the method section. We confirm that this choice is appropriate for this study.

## Results

A total of 28 women accepted to participate in the project and were consecutively allocated into three groups. Out of 28 registered women, 22 (79%) started the pathway. Characteristics of participants are reported in Table 5. The age range and the median time from infertility diagnosis were higher in group A than in the other groups (Table 5). Among women actually undergoing ART, three became pregnant during the project, and two left the pathway (groups A and B).

**Table 5:** Characteristics of included women.

	Overall (*n* = 22)	Group A (*n* = 7)	Group B (*n* = 8)	Group C (*n* = 7)
Median age (years) at enrollment (min–max)	36 (28–53)	36 (28–53)	36 (32–38)	37 (33–40)
Female infertility, *n* (%)	16 (80)	4 (67)	6 (86)	6 (86)
Male infertility, *n* (%)	4 (20)	2 (33)	1 (14)	1 (14)
Have children (*n*)	1	0	1	0
Median time (months) from infertility diagnosis (min–max)	24 (2–72)	66 (32–41)	18 (5–72)	24 (2–48)
Active phase of ART, n***	7	3	2	2

Information about the diagnosis of infertility was not available in two cases, whereas in five cases duration from diagnosis was missing. Women not in active phase of ART, had already gone through the process, or were waiting to go through active phase.

*** Procedures: ovum pickup (1); embryo transfers (2); unspecified (4).

The starting questionnaire was answered by 82% of women, and their expectations are reported in Table 1. The three groups started the same pathway in the same period. Six women withdrew from the project after the first meeting and two after the second meeting; three women entered the project at the second meeting. The withdrawal was due to organization (*n* = 1) and inadequate phases in the ART process (*n* = 7). In the synchronous phase, 57% of meetings were attended; 50% of suggested prompts were answered in the asynchronous phase. The final tale was shared on the platform by 45.4% of participants.

### Emerging contents and narrative

Each group chose a name to indicate the journey the women were starting together: Looking for Lost Harmony (group A), the Country of the Magic Forest (group B), or the Country of Little Blooming Masks (group C).

Several descriptions were given for the country of narrating masks: a fantasy and mysterious place with shady characters, a place that used to be quiet and has been disrupted, the place of awareness, the place of still and uncertain time where one can easily get lost (the most frequent description): “This is how I imagine the Country of narrating masks…time is flowing…each time one waits for weeks months…each failure is the same.”

The Talent mask was used to get in touch with personal resources: capacity to start again and not surrender after a failure, capacity to orienteer and choose their own path, capacity to struggle and determination, and capacity to take adverse even slightly. All women felt that the group support was a fundamental and useful resource to enhance positive emotions, share the same situations and difficulties, comfort each other, experience quiet through shared pleasure and enjoyment, and regain confidence in the pathway.

The Restless Being was the most relevant character for all the participants. It was described as a contingency, confusion, dissonance, others’ judgment, fear of failing, obsessive thought, anger, and injustice. It was very important to be able to contact, describe and represent the chaotic experience induced by the Restless Being’s will to rule the women’s interior world. This activity enabled women to put a face to their suffering and change it through dialogue with their own resources. Indeed, participants represented the meeting of Talent and Restless Being, expressing characteristics and needs of both, resulting in a changed perspective. “After we discovered that Restless Being is not so bad, on the contrary, it is almost a partner, our outlook on life was changed….” “[Restless Being] surrenders and gives us the narrating mask of the Queen.” “Try and move a little; things may look completely different from a new perspective.”

Having back the Queen mask was a new harmonic equilibrium obtained by the support of a shared pathway. The conclusion of one of the final videos was: “They walked a long time sheltering the mask with all their might until they met the Queen and gave her back what was rightfully hers…once she put on the mask, she had her light again, and she smiled” (Video a, 2021; Video b, 2021; Video c, 2021).

### Outcomes

The final evaluation questionnaire was answered by 54.5% of the participants. Compliance with the study and feasibility and utility scores given by participants are reported in Table 6. Overall, scores of feasibility and utility are >4 (5 is the highest score) in 75% of cases. Individual opinion seems to be related to continuity of participation in the group. The utility was scored higher by women who attended the whole pathway, with a supportive and open debate group. The lowest score was given by a woman in group A who attended the whole program but could not have a stable and continuous group; nevertheless, she gave a positive score for feasibility.

**Table 6:** Participants’ overall evaluation: compliance and feasibility/utility scores (1 = min, 5 = max).

Group	Presence at meetings	Compliance to the synchronous phase	Compliance to prompts	Final Production	Compliance to the asynchronous phase	Feasibility score (mean values)	Utility score (mean values)
A	5	100%	14/14	Yes	100%	4	2.75
A	3	60%	7/14	No	50%	4.77	4
B	5	100%	14/16	Yes	87.50%	4.23	5
B	3	60%	13/16	Yes	81.25%	5	5
B	5	100%	16/16	Yes	100%	4.67	3.5
B	5	100%	16/16	Yes	100%	3.23	4.25
C	5	100%	16/16	Yes	100%	4	4.25
C	5	100%	16/16	Yes	100%	4	4.25
C	2	40%	3/16	No	18.75%	3.37	3.75
C	5	100%	16/16	Yes	100%	4.4	4.5
C	4	80%	12/16	Yes	75%	4.5	4.25
C	2	40%	9/16	No	56.25%	3.37	4

Some special issues in the final questionnaire were associated with low scores in group B: the desire to have personal feedback (*n* = 1) and a technical problem in uploading the final tale (*n* = 1).

Based on feedback from the participants along the pathway, the main areas of satisfaction were those of creativity, with an increased artistic expressivity and dramatic ability, resulting in a declared enhancement of self-confidence.

The overall mean scores of clinical parameters (climate, cohesion, alliance, interdependence, defense, therapeutic benefit) given by researchers to groups are reported in Table 2. Eighty percent of meetings had at least 35% of participants and were valuable. The assessment rating is generally positive and consistent within the group, with a few exceptions: group C had a high “identification” (with the group) and a low “autonomy” (individual autonomy) score; group B had a low “realism” with a high “symbolize” (capability to symbolize) score.

Table 7 shows the overall mean scores of clinical parameters given to groups B and C in each meeting and the focus group. Group A was not valuable in the whole pathway. Group B obtained better scores in meeting 4. During this meeting, participants used the Queen mask to send themselves relevant messages, such as the importance of sharing to regain confidence in their internal resources and discover new perspectives in the face of problems. The score of therapeutic benefits progressively increased in the group, in which the relationship of components strengthened over time.

**Table 7:** Mean scores of clinical parameters given to groups B and C by researchers in each meeting, and the focus group.*

	Group B					Group C				
	1° meeting	2° meeting	3° meeting	4° meeting	Focus Group	1° meeting	2° meeting	3° meeting	4° meeting	Focus group
Climate^	4.5	5	5	5	4.5	3.5	5	4.5	3.5	4.5
Cohesion^	3.67	2.67	4	5	4	2.33	5	4	4	4
Alliance^	3.4	3.4	2.8	4.8	4	2	4.2	4	4	4
Interdependence^	4.33	3.4	4	4.33	4.33	3	3.67	3.33	3.5	4.33
Defense^§^	1.25	1.13	1.13	1.25	1.25	1	1.25	1.13	1.25	1.13
Therapeutic Benefit^	3	3.38	3.5	4.13	4.25	2.63	3.5	3.5	3.13	3.75

§ Lower values stand for positive meaning.

^ Higher values stand for positive meaning.

* Group A was not evaluated because participants met only two times out of the scheduled four meetings.

On the contrary, the mean scores of group C were discontinous, probably due to the withdrawal of two participants. Nevertheless, the other participants could obtain therapeutic benefits (data not shown). “Interdependence” got its highest scores in the focus group, showing that participants desired to maintain their relationship. Both groups decided to keep in touch through independent tools such as public social media to continue the experience after the Integrated Dramatherapy pathway.

All groups received an applicability score related to ease of use of the platform of 5/5. Limited to groups C and B, the remaining applicability items (Table 4) received a score of 5/5.

## Discussion

Although the interest in arts in care pathways is increasing, there is a need to find a shared methodology and validated interventions (Fraser and Al Sayah, 2011). This pilot study evaluated the applicability of Digital Integrated Dramatherapy in psychological support to women undergoing ART to foster studies in a clinical setting. From a systematic review of the literature, the authors found no previous published studies, including the application of Art Therapy or Dramatherapy in the same setting. This prevents comparisons and limits citation of literature in this discussion. However, several studies highlighted the effectiveness of Dramatherapy in different areas of health (schizophrenia, eating disorders, and oncological diseases) involving a target population of a wide range of ages and characteristics (Mazor, 1982; Irwin, 1986; Chasin et al., 1989; Bielańska et al., 1991; Ruddy and Dent-Brown, 2007; Knox and Svendsen, 2015; Keisari and Palgi, 2017; Purrezaian et al., 2020). A therapeutic intervention, which integrates life-review narrative together with drama therapy in older adults, was effective in increasing self-acceptance, relationships with others, a sense of meaning in life, and a sense of successful aging; it also decreased depressive symptoms (Keisari and Palgi, 2017). In a study involving hospitalized adolescent patients affected by eating disorders, theater workshop was useful in decreasing defense mechanisms, allowing a patient-focused approach, mitigating specific symptoms, and improving the quality of life during the hospital stay (Pellicciari et al., 2013).

However, the above-mentioned studies mainly refer to the use of the theatrical medium as a valid tool for therapeutic intervention. In the innovative Integrated Dramatherapy methodology, the use of artistic languages is combined with psycho-corporeal techniques, with the aim of bringing participants to the construction of a metaphorical path, integrating bodily movements, pictures research and production, figurative and plastic creation, reflective and creative writing, dramatization and verbalization. It draws on the dramatic play paradigm, devised by Sue Jennings, according to which there is a continuity between embodiment, projection and role, taking advantage of the possibility of using the three phases in an integrated and simultaneous way (Jennings, 1998). It is also based on the concept of ‘embodied metaphor’ (Milioni, 2008) and studies related to the Transitional Object and the Creative Act” (Modell, 1970).

Digital platforms have been demonstrated to be suitable for narrating in care pathways and remote psychological support; such tools have been extremely useful during the COVID-19 pandemic (Giusti et al., 2020; Cercato et al., 2022a,b). Expressly developed tools seem necessary for this use, and the operators should choose settings and functions according to the type of intervention. In addition, good compliance with privacy rules is required. Public social networks are unsuitable for personalized narratives and are not designed for data protection.

Participants assessed the good feasibility and utility of the method, both in the synchronous and the asynchronous phases, although continuity and persistence of the group could affect outcomes. The group has a fundamental role in our study: the participants were supportive, and therapeutic benefits were due to strengthening and resilience obtained through a dialogue with other women.

Participants shared their experience in ART at the beginning of the pathway through a multimedia narration (with photos, images, and music). This step was very important to promote confidence in the group and mirroring each other. Moreover, it was a common starting point for the symbolic journey into self-discovery and to develop new perspectives. Using adequate metaphors, the participants could move from the narration of the ART pathway to creative and corporeal expression. This process helped them talk about their painful experience and find new ways and solutions.

The study showed that group support might help women face very difficult emotions by promoting creativity and internal resources. Group support has a pivotal role in strengthening and increasing the resilience of women. The participants’ final assessment showed that the expectations declared at the beginning of the study had been met. Women had become aware of themselves and their emotions by sharing similar experiences with other women.

The assessment of the researchers confirmed the role of the group. A progressive increase of confidence in one’s resources was observed in group B, with the desire for new possibilities and self-care. A progressive increase was also observed for benefit scores of self-revelation, self-understanding, interpersonal learning, and insight. Despite withdrawals during the study and discontinous participation, Group C had a good cohesion. Indeed, mirroring and resonance had their highest scores in the focus group. Acceptance of differences showed many common points and great emotion in meetings.

The digital setting was also suitable for regaining the creativity of this population and helped all women participate, overcoming access barriers and fears of social contacts during the pandemic period.

The study had some limitations. The ART phase must be adequate to allow participation in DID, so as to ensure continuity in the pathway. Active phases of ART (pick up and transfer) were the main causes of withdrawal, and the inconsistency of participation affected the group cohesion. We acknowledge that this limitation may be relevant for the evaluation of utility. A second limitation was the disparity in the time from the diagnosis of infertility, the time already spent in the ART process, and ART outcomes. Varying and ambiguous expectations made the management of the group difficult. An example is a 53-year-old woman who had painfully experienced ART 10 years before and who shared, after the second meeting, some tales she had previously written about her story. She later withdrew because her current position was too different from the other participants “I feel I am stealing time to a dream no longer belonging to me.”

Furthermore, the digital pathway needs some improvement. The time interval between prompts must be scheduled regularly so that 15 multimedia prompts may be delivered. This latter is the number of prompts that were observed to be suitable to gradually draw participants from the realistic story of their ART experience to the country of narrating masks, showing they had learned a metaphoric language.

The digital environment provides intimacy and guarantees anonymous participation or a progressive, modulated revelation of one’s identity when necessary. A blended method, which alternates in-person meetings and asynchronous phases on the platform, could be explored. It could maintain the experience of workshops for longer periods of time, while a personal elaboration of prompts could be managed between meetings.

## Conclusion

The study suggests the applicability of Digital Integrated Dramatherapy to the psychological support of women undergoing ART. Video chat provided the experience of many perceptive, corporeal and expressive modalities. The asynchronous phase allowed the opportunity to obtain audio and images from the Internet to make the narrative richer and more expressive. Further studies are needed to validate the methodology in a clinical setting, possibly using a blended pathway with personal meetings and individual digital works.

## Data availability statement

The original contributions presented in the study are included in the article/supplementary material, further inquiries can be directed to the corresponding author.

## Ethics statement

Ethical review and approval was not required for the study on human participants in accordance with the local legislation and institutional requirements. The patients/participants provided their written informed consent to participate in this study.

## Author contributions

MC, SP, CC, and REN: study planning and article setting up. SP and RP: study conduction, sample collection, and texts analysis. CG and CC: patients recruitment and engagement. MC and IT: statistical analysis and results presentation. All authors contributed to the article and approved the submitted version.

## Funding

The study received an unconditional grant from IBSA Foundation for scientific research, Collina d’Oro, Switzerland. This work was financially supported through funding from th institutional “Ricerca Corrente” 2022 granted by Ministry od Health.

## Conflict of interest

CG and CC were employed by Digital Narrative Medicine (DNM).

The remaining authors declare that the research was conducted in the absence of any commercial or financial relationships that could be construed as a potential conflict of interest.

## Publisher’s note

All claims expressed in this article are solely those of the authors and do not necessarily represent those of their affiliated organizations, or those of the publisher, the editors and the reviewers. Any product that may be evaluated in this article, or claim that may be made by its manufacturer, is not guaranteed or endorsed by the publisher.
